# Incorporation of Local Structural Preference Potential Improves Fold Recognition

**DOI:** 10.1371/journal.pone.0017215

**Published:** 2011-02-18

**Authors:** Yun Hu, Xiaoxi Dong, Aiping Wu, Yang Cao, Liqing Tian, Taijiao Jiang

**Affiliations:** 1 National Laboratory of Biomacromolecules, Institute of Biophysics, Chinese Academy of Sciences, Beijing, China; 2 Graduate University of Chinese Academy of Sciences, Beijing, China; University of Queensland, Australia

## Abstract

Fold recognition, or threading, is a popular protein structure modeling approach that uses known structure templates to build structures for those of unknown. The key to the success of fold recognition methods lies in the proper integration of sequence, physiochemical and structural information. Here we introduce another type of information, local structural preference potentials of 3-residue and 9-residue fragments, for fold recognition. By combining the two local structural preference potentials with the widely used sequence profile, secondary structure information and hydrophobic score, we have developed a new threading method called FR-t5 (fold recognition by use of 5 terms). In benchmark testings, we have found the consideration of local structural preference potentials in FR-t5 not only greatly enhances the alignment accuracy and recognition sensitivity, but also significantly improves the quality of prediction models.

## Introduction

Modeling of protein structures based on structure templates found from experimentally determined structures, called template-based modeling (TBM), is currently the most effective way to build a 3-D structure for a protein of unknown structure. To build a structure model for a target protein sequence, the TBM process consists of four major steps: identification of structural templates, alignment of target sequence to structural templates (or sequence-structure alignment), model building, and model quality evaluation. The first two steps are the key steps in the TBM process, improvement of which can greatly improve the quality of the final predicted model [1], [2], [3], [4], [5], [6], [7], [8], [9], [10], [11], [12], [13]. For target sequences with high sequence similarity to those of structure templates, the structural templates can be easily identified and the target sequences can be reliably aligned to the structural templates by those methods that use sequence information alone such as PSIBLAST [14] and HMMER [15]. However, for target sequences with low sequence similarity, the reliable identification of structural templates and accurate sequence-structure alignment requires a much more complex process called threading or fold recognition that integrates many other types of information with sequence profile information.

The secondary structure information is probably the most popular one that has been integrated with sequence profile information in most of the existing fold recognition methods [9], [10], [11], [16], [17], [18], [19]. Other types of structural information such as contact information, solvent accessibility, predicted backbone torsion angles and structure profiles have also been explored to improve the accuracy of fold recognition [20], [21], [22], [23], [24], [25]. Arguably, the integration of a proper type of structural information can significantly improve fold recognition, particularly for those target sequences with low sequence similarity to structural templates of similar fold.

In this work, we introduce another type of structural information, local structural preference information, in fold recognition. The structure preferences of 3-residue and 9-residue fragments were derived as potential-like terms from known structures. We have shown that integration of these terms with the three widely used information, sequence profile, secondary structure and hydrophobic score allows us to develop an effective fold recognition method, called FR-t5, an abbreviation of fold recognition with 5 terms.

## Results

### Overview of the FR-t5, a novel fold recognition approach by considering local structure preference potentials (LSPPs)

We have derived local structural preference potentials (LSPPs) to capture the structure preference of sequence fragments of short length. Fragments of 3- and 9-amino acids are considered in our work. To calculate the 3-residue and 9-residue LSPPs, we first divide the conformers of 3-residue and 9-residues fragments into a number of bins, then compute the distribution of these binned conformers among known structures (Details see Methods). By combining 3-residue and 9-residue LSPPs with the three widely used information, sequence profile, secondary structure and hydrophobic score, we further develop a new threading algorithm called FR-t5. In FR-t5, dynamic programming (DP) [26], [27] is used to make alignments between the query and the templates. Then the templates are selected to build the structure models for the query protein sequence using MODELLER [28]. A detailed description of the method is given in Methods.

In the following results, we will first show based on different tests that the incorporation of LSPPs indeed improves the fold recognition of FR-t5 in both the threading alignment and the sensitivity of fold recognition by comparing to the method without considering LSPPs which we called as FR-t3 for convenience. Then, we will compare the FR-t5 to the state-of-the-art fold recognition methods. Finally, we will demonstrate the performance of FR-t5 in the recent CASP9 of 2010. The consideration of LSPPs has enabled us to develop an effective fold recognition approach.

### LSPPs improves the threading alignment in FR-t5

To test whether the incorporation of LSPPs improves the alignment accuracy, the performance of FR-t5 was evaluated by comparing to FR-t3 on two datasets: SALIGN [29] and MUSTER190 [19]. The SALIGN dataset consists of 200 pairs of structurally similar proteins with 65% of equivalent Cα atoms superposed within an RMSD of 3.5 Å. But the sequence similarity of these SALIGN protein pairs is low, ∼20% sequence identity on average. The Muster190 dataset contains 190 protein pairs whose structural similarities are indicated by SCOP hierarchical structure classification, 120 of them having same folds but in different superfamilies and 70 of them belonging to same superfamilies but not same family. The structural alignments used as gold standards were carried out by the TM-align program [30]. The MUSTER190 dataset could be more difficult to align than SALIGN, because the protein pairs of MUSTER190 (average TM-score  = 0.536) are less structurally similar than those of SALIGN (average TM-score  = 0.653).

To compare the alignment accuracy of FR-t5 and FR-t3 on the two datasets, for each protein pair, we align the query with its template using the threading program FR-t5 and FR-t3, respectively. The alignment accuracy is calculated as the percentage of correctly aligned positions by comparing to the gold standards generated by the TM-align program (see Table 1).

**Table 1:** The alignment accuracies for FR-t5 and FR-t3 on SALIGN and MUSTER190 datasets.

Method	SALIGN	MUSTER190
FR-t3	57.1±0.14%^a^	35.1±0.20%
FR-t5	58.9±0.16%	36.0±0.19%

a Mean value and the standard error (estimated by bootstrap simulation on 10,000 re-sampling of the dataset).

Table 1 summarizes the alignment accuracies of FR-t5 and FR-t3 on both datasets. Obviously, FR-t5 achieves better alignments than FR-t3 (58.9% vs 57.1% on SALIGN and 36.0% vs 35.1% on MUSTER190). These tests demonstrate that the consideration of LSPPs in fold recognition can improve the alignment accuracy.

### LSPPs improves fold recognition sensitivity in FR-t5

To further investigate whether the consideration of LSPPs is able to improve the fold recognition sensitivity, we compared FR-t5 with the method without considering LSPPs, FR-t3, on the Lindahl dataset [31] which is a widely used dataset for benchmarking the sensitivity of other threading programs [3], [6], [7], [10], [11], [31], [32], [33]. The Lindahl dataset includes 976 proteins, of which 555, 434 and 321 proteins have at least one match with the others in the dataset at the family, superfamily and fold levels, respectively. To evaluate the contribution of LSPPs in fold recognition sensitivity by comparing FR-t5 to FR-t3, each protein was aligned with the other 975 proteins. The fold recognition sensitivity is measured as the percentages of the true hits identified as the first rank or as one of the top five ranks (see Table 2).

**Table 2:** The benchmarking of the sensitivity of FR-t5 and FR-t3 on Lindahl dataset.

Method	Family (%)		Superfamily (%)		Fold (%)		
	Top1	Top5	Top1	Top5		Top1	Top5
FR-t3	81.6	89.7	49.1	64.7		29.6	58.4
FR-t5	84.0	90.2	54.0	71.9		35.0	65.5

As shown in Table 2, the FR-t5 outperforms FR-t3 in Top1 by 2.4%, 4.9%, and 5.4%, at the level of family, superfamily, and fold, respectively, indicating that the consideration of LSPPs can improve the sensitivity of fold recognitions at all SCOP levels. But compared to the improvement at family level (84.0% versus 81.6% in Top1 and 90.2% versus 89.7% in Top5), the improvements at the superfamily level (49.1% versus 54.0% in Top1 and 64.7% versus 71.9% in Top5) and the fold level (29.6% versus 35.0% in Top1 and 58.4% versus 65.5% in Top5) are even more significant. This shows the advantage of the incorporation of LSPPs for fold recognition in its ability to significantly improve fold recognitions for proteins sharing low sequence similarity.

### The consideration of LSPPs in FR-t5 significantly improves the quality of structure modeling in CASP8 test set

To gain more comprehensive insights into the contribution of LSPPs in fold recognition, the methods with (FR-t5) and without (FR-t3) consideration of LSPPs were more rigorously compared by applying them to find structure templates and make structure prediction for the CASP 8 targets [34]. In CASP 8, 164 domains from 121 target proteins to be predicted were used to evaluate the server prediction performance [35]. Of the 164 domains, 13 were defined as free modeling (FM) targets and 154 as template-based (TBM) targets (including 3 FM targets). Of the 154 TBM targets, 50 were further defined as the high-accuracy (TBM-HA) targets. The above classification was based on sequence and structure similarity [35]. To ensure a blind prediction, we only used the non-redundant (NR) sequence database (ftp://ftp.ncbi.nih.gov/blast/db) and PDB database [36] generated before the start of CASP8. The prediction performance is evaluated by the TM-score of the first model and Top 5 models.

As shown in Table 3, when the first models for all 164 targets were considered, FR-t5 outperforms FR-t3 in both TBM targets and FM targets by an improvement of TM-score about 1.9% on average. The improvement of FR-t5 over FR-t3 is more significant for the difficult FM targets of no detectable templates: a 12.5% (0.025/0.2) increase of TM score. While for the easy 50 TBM-HA domains, there is no significant improvement, emphasizing the contribution of LSPPs to the structure prediction beyond sequence similarity.

**Table 3:** The comparison of FR-t5 and FR-t3 on CASP8 test set.

Method	ALL^a^		TBM^b^		TBM-HA^c^		FM^d^	
	Top1^e^	Top5^f^	Top1^e^	Top5^f^	Top1^e^	Top5^f^	Top1^e^	Top5^f^
FR-t3	0.629	0.661	0.660	0.691	0.837	0.852	0.200	0.258
FR-t5	0.641	0.673	0.670	0.700	0.837	0.862	0.225	0.277

a All 164 target domains(there are 3 overlap targets between TBM and FM categories).

b 154 TBM target domains.

c 50 TBM-HA target domains.

d 13 FM target domains.

e The average TM-scores for Top1 models of the two methods are given.

f The average TM-scores for Top5 models of the two methods are given.

### Comparison with other methods

As shown above, the incorporation of LSPPs can significantly improve both alignment accuracy and sensitivity of fold recognition. Here we ask whether FR-t5 which simply incorporates local structural preference information into the three widely used terms (sequence profile, secondary structure and hydrophobic score) can achieve a satisfactory performance that is comparable to the existing popular fold recognition programs. In developing fold recognition methods, the SALIGN dataset [29] and Lindahl dataset [31] have been widely used to test alignment accuracy and fold recognition sensitivity, respectively. In order to compare FR-t5 with the existing popular fold recognition methods directly, we carried out the tests based on these two datasets that had been previously used to test or develop these existing methods.

Based on the dataset SALIGN that was used to assess the performance of BLAST [37], COMPASS [38], SALIGN [29], SPARKS [7], SP3 [9] and UNI-FOLD [39], we compared the alignment accuracy of FR-t5 with the alignment accuracies of these methods reported in the literature [39]. As shown in table 4, FR-t5 is slightly better than UNI-FOLD, the best of these methods (58.9% vs 57.4%).

**Table 4:** The alignment accuracy (%) of FR-t5 on the SALIGN test data.

Methods	Acc
FR-t5	58.9
BLAST	26.1
COMPASS	43.2
SALIGN	56.4
SPARKS	53.1
SP3	56.6
UNI-FOLD	57.4

Since the programs BLAST, COMPASS, SALIGN, SPARKS, SP3, UNI-FOLD have all been tested on the SALIGN test data previously, for comparison, their results were taken from the previous studies: BLAST, COMPASS, and SALIGN from Marti-Renom et al [29], SPARKS and SP3 from Zhou and Zhou [9], and UNI-FOLD from Poleksic and Fienup [39].

Based on the Lindahl dataset, we also compared the fold recognition sensitivities between FR-t5 and the existing 9 threading methods that demonstrated good performance in previous CASPs, namely SAMT98 [40], FUGUE [3], RAPTOR [6], SPARKS [7], HHpred [18], FOLDpro [32], SP3 [9], SP4 [10], SP5 [11]. Table 5 shows that in terms of fold recognition sensitivity, FR-t5 is comparable to the best of these existing 9 threading methods in finding structural templates for proteins with a wide range of sequence similarities to their template structures (from the family level to fold level).

**Table 5:** The comparison of FR-t5 with other methods for fold recognition on the Lindahl benchmark.

Methods	Family (%)		Superfamily (%)		Fold (%)	
	Top1	Top5	Top1	Top5	Top1	Top5
FR-t5^a^	84.0	90.2*	54.0	71.9*	35.0	65.5*
SAMT98^b^	70.1	75.4	28.3	38.9	3.4	18.7
FUGUE^b^	82.2	85.8	41.9	53.2	12.5	26.8
RAPTOR^b^	75.2	77.8	39.3	50.0	25.4	45.1
SPARKS^b^	81.6	88.1	52.5	69.1	24.3	47.7
FOLDpro^b^	85.0*	89.9	55.5	70.0	26.5	48.3
HHpred^c^	82.9	87.1	58.8	70.0	25.2	39.4
SP3^c^	81.6	86.8	55.3	67.7	28.7	47.4
SP4^c^	80.9	86.3	57.8	68.9	30.8	53.6
SP5^c^	81.6	87.0	59.9*	70.2	37.4*	58.6

a this work.

b, c Results are cited from from Refs [32] and [11], respectively.

*The best results are marked by asterisk.

### Participation of FR-t5 in the recent CASP 9

Our newly developed FR-t5 has participated in the recent CASP9 of 2010 under the name of Jiang_THREADER. As a server group, Jiang_THREADER made structure prediction for all the 147 domain targets provided by CASP9. Based on the evaluation, our program Jiang_THREADER was ranked 24^th^ among all 81 structural modeling programs (http://predictioncenter.org/casp9/CD/data/html/groups.2.html), demonstrating the relative good performance of our FR-t5 in structural modeling, which is comparable to most of the state-of-the-art structural modeling programs.

**Figure 1:**
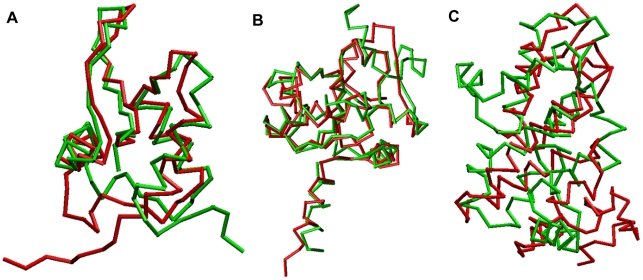
Modeled structures for three CASP9 targets, T0549, T0592 and T0553, by FR-t5. (a) The superposition between the native structure of T0549 (green) and the top1 model (red) predicted by FR-t5. (b) The superposition between the native structure of T0592 (green) and the top1 model (red) predicted by FR-t5. (c) The superposition between the native structure of T0553 (green) and the top1 model (red) generated by FR-t5.

The prediction results from all participated methods have been released online (http://predictioncenter.org/download_area/CASP9/server_predictions/), allowing us to make comparisons based on individual predictions. Here we would like to show some successful examples predicted by FR-t5 (See Figure 1). One example is T0549 of 84 AA, the FR-t5 predicts the model with a TM-score of 0.662 which has the best performance among all prediction methods;Another example is T0592 of 144 AA, the FR-t5 predicts the model with a TM-score of 0.771, which comes next to Raptor (with a TM-score of 0.789). For the more difficult target T0553 of 141 AA, FR-t5′s prediction is the best among the fold recognition methods, which predicted a model with a TM-score of 0.332 that is comparable to the de novo prediction method BAKER-ROSETTASERVER with a TM-score of 0.331. We note that it is hard to do a fair comparison with other prediction methods based on the prediction models submitted to the CASP prediction center. First, as pointed out by Wu and Zhang [19], the threading performance is usually sensitive to the template library which varies greatly between different methods. Second, in CASP, some predictions could combine several threading methods (so called meta-threading methods) [5], [41], [42], [43], integrate multiple templates [44], [45], [46], [47], perform optimizations such as all-atom refinement [48], [49] and employ ab initio prediction when the correct templates are ambiguous [45], [48], [50]. However, our FR-t5 prediction does not incorporate the results of other methods of same kind or perform any further refinement. Nonetheless, the relative good performance of FR-t5 in CASP9 has demonstrated its potential application to structure modeling.

## Discussion

In this work, we have developed a new threading method FR-t5 by combining the information of local structural preference for 3-residue and 9-residue fragments with sequence profile, predicted secondary structure, and hydrophobic scoring. The incorporation of the two new terms is intended to capture the local structure stiffness when the template structure is aligned with the query sequence. To explore the effects of the two new terms on the improvement of fold recognition, the FR-t5 method is compared with the method FR-t3 that only considers sequence profile, predicted secondary structure and hydrophobic scoring. Based on testings on three public benchmarks, we have shown that the incorporation of the two terms improves both the alignment accuracy and recognition sensitivity in fold recognition. Moreover, when testing on the CASP8 targets, we found that incorporation of the two new terms can significantly improve the structure modeling for the targets of low similarity, with an improvement of TM-score of 12.5% for the 13 hard targets.

Many pioneering studies have analyzed the characteristics of recurring local structural fragments and their mappings to local sequence properties [51], [52], [53], [54], [55], [56], [57], [58], [59], [60]. The mappings between local sequence and structure have been used to improve protein structure modeling. The Rosetta program [59] uses the information of 3-residue and 9-residue fragments for de novo protein structure prediction and refinement of protein prediction models. In the SP3/SP4/SP5 [9], [10], [11] and MUSTER [19], the local structural profiles derived from 25 top fragments in the comparison of the 9-residue fragment with all same-size fragments in the structural database have also been shown to contribute much to the good performance of fold recognition. Recently, Zhou and Skolnick showed that use of fragment comparison and template comparison which provide local and global quality evaluation of the prediction model, respectively can better rank and assess the prediction model [24]. These studies have demonstrated the direct use of local sequence and structure mappings in terms of fragment library to improve protein structure modeling. In our study, inspired by the work of Shakhnovich group that used a local sequence-energy term for protein structure de novo prediction [61], we have derived a statistics-based local structural preference potential (LSPP) for 3-residue and 9-residue fragments for fold recognition. Indeed, the integration of 3-residue and 9-residue LSPPs into the three widely used information, sequence profile, secondary structure and hydrophobic score has led us to develop the effective fold recognition program, FR-t5. We believe the development of the local structural preference potential will be of great benefits for application, because it is independent of databases and requires no structural comparison which is computationally expensive. Therefore, the local structural preference potential we developed can be easily incorporated into other threading methods.

Consideration of individual terms that are independent is very important for development of an effective knowledge-based scoring function. In our work, we have considered two types of structural information. One is the secondary structure information and the other is local structural preference information. Although the two types of structural information can be highly correlated, they are different, which can capture different aspects of structural feature. The secondary structure term is intended to capture the secondary structure propensity of a residue, which is based on three crude categories: alpha helix, beta sheet and coil regions. While the LSPPs used in our study is able to capture more detailed local structural conformation at short fragment level. In our testing, we found that the incorporation of LSPPs significantly improve the performance, suggesting the complementary nature of the two types of structural information in fold recognition.

Although we have shown that consideration of local structure information in potential-like forms has significantly improved fold recognition. There is still much room to improve. First, the fragment sizes have not been extensively explored. In our study, for simplicity, we only attempted fragments of 3-residue and 9-residue. Second, since different representations of the structural fragments can reveal different features of the local structures, finding better way of structure representation could dramatically improve fold recognition. Lastly, a more systematic and comparative analysis is needed to look for discretization of the local structural space, which will generate more appropriate bin numbers for the improvement of fold recognition. Despite this, our consideration of local structural preference information has led us to develop an effective fold recognition method, FR-t5, which can achieve a comparable performance to the existing well-established threading methods.

## Methods

### Local Structural Preference Potential of 3-residue Fragments

**Figure 2:**
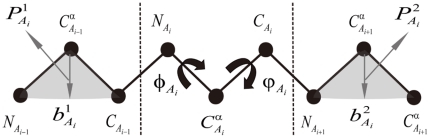
A Schematic Diagram of Spatial Representation and Conformational Constraints of a 3-residue Fragment. The bold letters 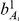 and 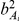 denote the bisecting vector of 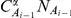 and 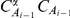, the bisecting vector of 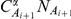
and 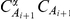, respectively. 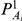 and 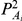 denote the vectors in planes defined by three backbone atoms (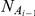,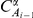, and 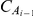), and (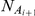,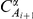, and 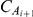), respectively.



The local structural preference potential of the 3-residue fragment is computed by following Yang et al's method [61] with adaptation. Let us suppose a 3-residue fragment that consists of three amino acids 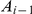, 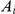, and 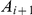, the four variables 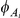,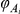,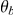 and 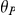 are used to represent the conformation space of the 3-residue fragment, where 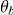 is the angle between 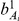 and 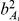, 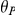 is the angle between 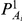 and 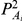, respectively (see Figure 2). The width of bins was 60°, 60°, 30°, 30° for 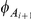,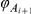,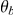 and 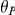, respectively. The potential of the 3-residue fragment 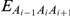 is obtained from the Potential Database (see below) by:

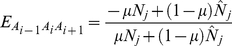
(1)where 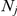 and 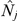 are the number of observations in the *j*-th bin and the total number of observations not in the *j*-th bin, respectively. The normalization process requires the careful choice of the value of µ (0<µ<1) to balance the contribution of the positive counts 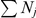 and the negative counts 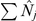 for all the bins in the database. Because the total number of observations not in the j-th bin, 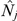 is always far larger than number of observations in the j-th bin, 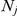, a big weight (µ>0.5) should be given to the positive counts. The value of µ should be chosen to make 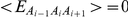 (net interaction energy, i.e., the average of energies for the bins that have at least one positive count.). In the case of 3-residue fragment, the value of 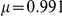 is chosen to make the net interaction zero. The potentials of all 8000 3-residue fragments over the binned conformers constitute an energy table.

In threading, we assume a 3-residue fragment to adopt its template conformation, then its local structural preference potential 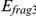 can be obtained from above energy table.

### The Local Structural Preference Potential of 9-residue Fragment

**Figure 3:**
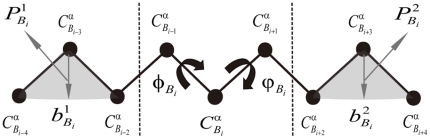
Coarse-grained Structure Model of a 9-residue Fragment. The coarse-grained structure of a 9-residue fragment consists of nine points, each of which represents an amino acid and is denoted as the Cα atom of the residue. A link between two Cα atoms is a virtual bond that connects the two residues. Thus, the description of the coarse-grained structure of a 9-residue fragment follows that for 3-residue fragment (see Figure 2). The bold letters 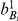, 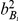 denote the bisecting vector of 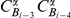 and 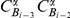, the bisecting vector of 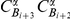 and 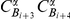, respectively. 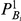, 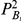 denote the vectors in a plane defined by three continuous 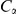 atoms (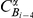,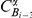, and 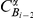), and (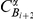,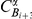, and 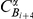), respectively.



The local structural preference potentials of a 9-residue fragment describe the statistical distributions of its binned conformers. Given the myriad of conformers of 9-residue fragments, to avoid the undersampling issue, we introduce a coarse-grained model described as follows: First, to reduce the sequence space of 9-residue fragments, the 20 amino acids were re-represented as three alphabets based on their hydrophobic-polar properties: H for hydrophobic residues F, W, Y, C, M, I, L and V; N for neutral residues A, G, T, S and P; and P for polar or hydrophilic residues N, Q, D, E, H, R and K [62]. Then, to decrease degrees of freedom in the conformation of a 9-residue fragment, each residue is represented by its 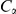. Supposing 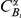 is the 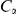 atom of the residue 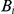 (where 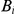 is the HNP type of a residue *i*), Figure 3 illustrates the coarse-grained model of a 9-residue fragment that centers on the residue *i*. In the coarse-grained model, the conformation of 9-residue fragment has same number of degrees of freedom as 3-residue fragment (comparing Figure 3 and Figure 2). Therefore, by following the conformation annotation of 3-residue fragment as shown in Figure 2, we introduce 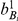 to represent the vector bisecting two vectors (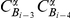 and 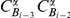) and 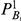 to denote a vector in a plane defined by three continuous 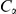 atoms (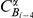,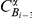, and 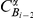). Therefore, the four variables 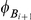, 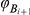,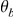, and 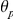 can also be used to describe the reduced conformation space of 9-residue fragment, where 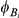 is the virtual dihedral angle defined by four continuous 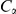 atoms (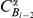,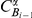,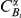, and 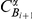); 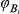 is the virtual dihedral angle defined by four continuous 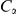 atoms (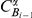,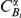,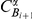, and 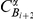); 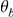 is the angle between 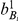 and 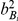; and 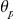 is the angle between 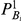 and 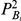. 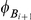, 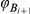, 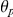, and 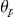 are further binned at intervals of 60°, 60°, 30° and 30°, respectively. The total number of bins is 3^9^*(360/60)*(360/60)*(180/30)*(180/30) ≈25.5 million, is greater than the number of 9-residue fragments (about 1.5 million) in the structural template database. Because most of bins are inaccessible due to the position features of the Cα atoms of the protein backbone [63], [64], the actual number of bins (i.e., the size of the energy table) is significantly reduced and thus the undersampling issue can be avoided. The structural preference potential of 9-residue fragment 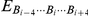 is obtained from the Potential Database (see below) by:

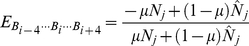
(2)where 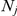 and 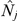 are the number of observations in the *j*-th bin and the total number of observations not in the *j*-th bin. The value of 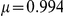 is chosen to make the net interaction zero.

In threading, we assume a 9-residue fragment to adopt its template conformation, then its local structural preference potential 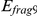 can be obtained from above energy table.

### The Potential Database

The parameters of local structural preference potentials for 3-residue and 9-residue fragments are derived from the PDB database released before CASP8 beginning date of May 3^rd^ of 2008. The non-redundant PDB library of sequence identity ≤30% was generated with PISCES [65]. The sequences that share sequence identity greater than 30% to the sequence in the training dataset ProSup [66] were further removed, resulting in 6298 sequences whose structures were determined by X-RAY with resolution higher than 3.0 Å. If there are any chain breaks in the fragment, the value of the energy is set to a reference value of 0.

### Scoring Functions

The local structural preference potentials for 3-residue (E*_frag3_*) and 9-residue fragment (E*_frag9_*) are combined with the three widely used terms, sequence profile (E*_seq,seq_*), secondary structure (E*_2nd_*) and hydrophobic score (E*_hydro_*) to make up a scoring function for template-based modeling. The shift constant (E*_shift_*) is introduced to avoid the alignment of unrelated residues in the local regions [19]. Thus, the score *E_(i,j)_* for measuring the extent/quality of alignment between the *i*th residue of a query sequence and the *j*th residue of a template sequence of known structure is a linear combination of the above five terms and the shift constant, which is given as follows:
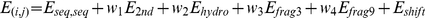
(3)where w_i_ are the weights of the equation, which were obtained by training the equation on the dataset ProSup (see below). The calculation of E*_seq,seq_*, E*_2nd_* and E*_hydro_* sees below. For simplicity, fold recognition or template-based modeling by using the scoring function with 5 terms is called FR-t5, while the method that uses the scoring function consisting of the three terms, sequence profile, secondary structure, and hydrophobic score is denoted as FR-t3.

### Sequence Profile, E_seq,seq_

For a given sequence, its sequence profile was built by using PSIBLAST [14] to search against the non-redundant (NR) sequence database. The PSIBLAST was run at e-value cutoff 0.001 with 3 iterations.

The term 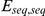 in Equation 1 is the sequence profile match score between query sequence and template sequence,which is computed as:
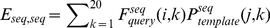
(4)where 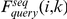 is the frequency of the presence of residue *k* of the template sequence at the position *i* of the query sequence, 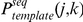 is the log-odds profile value (Position-Specific Substitution Matrix in PSIBLAST) of the residue *k* at position j of template sequence.

### Secondary Structure, E_2nd_

The term 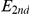 in Equation 1 is the match score between the predicted secondary structure of the query sequence and the observed secondary structure of the template structure, which is given below:
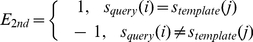
(5)where 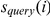 is the predicted second structure of the query sequence at position *i* and 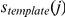 is the observed second structure of the template at position *j*. The secondary structures of query sequences are predicted by the program PSIPRED [67]. The secondary structures of template structures are assigned by DSSP [68]. The secondary structures are represented by three states, Helix (H), Strand (E), and Coil (C).

### Hydrophobic score, E_hydro_

The term 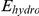 is the match score of the hydrophobic patterns between the query sequence and template sequence, which is given below:
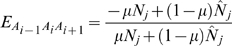
(6)where 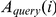 is the residue type of the query sequence at position *i* and 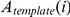 is the residue type of the template at position *j*. The hydrophobic scoring matrix is taken from Silva [23].

### The Gap Model

The gap model in the threading algorithm is an important factor that affects the alignment accuracy. Many different gap models were introduced previously. For example, SP3/SP4 [9], [10] and MUSTER [19] used a position-dependent gap penalty model which depends on the type of secondary structure. SP5 [11] used a profile-based gap model, which depends on the multiple sequence alignment made by PSIBLAST. More recently, Peng and Xu [69] used a more complicated gap model, which uses both context-specific and position-specific gap penalty. In our work, we employed the position-dependent gap penalty model in the dynamic programming, which operates as follows:

1. No gaps are allowed in the region where the predicted secondary structure and the secondary structure of the template are in the same state of helix or sheet;
2. The end gap penalty is neglected;
3. Affine gap opening (go) and gap extension penalties (ge) are applied to other regions.

### Dynamic Programming

We use the Needleman-Wunsch global alignment algorithm [26] to optimize the matching score between the query sequence and template structure based on Eq. (1) with the position-dependent gap penalty model described above.

### Parameterization of the Scoring functions

The parameters used in FR-t5 and FR-t3 were trained on the ProSup dataset [66] that consists of 127 protein pairs of significant structure similarity but of low sequence identity less than 30%. The alignments of these protein pairs are given by ProSup program and used as gold standards in the parameterization. There are 5 and 7 parameters (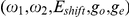, 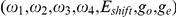), in FR-t3 and FR-t5, respectively, which were parameterized by following the same training procedure used by Zhou and Zhou [9]. In brief, to optimize the parameters, we maximized the number of matches between the gold standard alignment and the alignment made by the threading method.

### Template Selection

The template rankings are based on two normalized scores: Sn1 and Sn2. Sn1 is the raw score S normalized by the length of alignment including gaps between the query and template sequences. Sn2 is the raw score normalized by the alignment length excluding query ending gaps. To rank the templates of a query sequence, if the maximal Sn1 is greater than or equal to the maximal Sn2, they will be ranked by Sn1, and otherwise by Sn2.
